# The Effect of *Labisia pumila *var. *alata* on Postmenopausal Women: A Pilot Study

**DOI:** 10.1155/2012/216525

**Published:** 2012-05-31

**Authors:** Azidah Abdul Kadir, Nik Hazlina Nik Hussain, Wan Mohammad Wan Bebakar, Dayang Marshitah Mohd, Wan Mohd Zahiruddin Wan Mohammad, Intan Idiana Hassan, Norlela Shukor, Nor Azmi Kamaruddin, Wan Nazaimoon Wan Mohamud

**Affiliations:** ^1^School of Medical Sciences, Universiti Sains Malaysia, Kubang Kerian, 16150 Kelantan, Malaysia; ^2^School of Health Sciences, Universiti Sains Malaysia, Kubang Kerian, 16150 Kelantan, Malaysia; ^3^Department of Medicine, Universiti Kebangsaan Malaysia, Kuala Lumpur, 56000 Cheras, Malaysia; ^4^Institute for Medical Research, 50588 Kuala Lumpur, Malaysia

## Abstract

This is a randomized, double-blind, placebo-controlled study comparing the effects of a water extract of *Labisia pumila* var. *alata* at 280 mg/day with placebo, given for 6 months in postmenopausal Malay women. There were 29 patients treated with *Labisia pumila* and 34 patients in the placebo group. Menopausal symptoms were assessed at baseline and at 6 months. The blood pressure, body mass index, waist circumference, fasting blood sugar, lipid profile, and hormonal profile (follicle stimulating hormone/luteinizing hormone/estradiol) were measured during visits every two months. ANCOVA model analysis showed significantly lower triglycerides levels in LP subjects at 6 months after treatment as compared to placebo (1.4 versus 1.9 mmol/L; adj. mean difference 0.5, 95% CI: 0.02, 0.89 after adjusted for the baseline values, age, BMI, and duration of menopause placebo). Other parameters in both groups did not differ significantly. In conclusion, daily intake of *Labisia pumila* at 280 mg/day for six months was found to provide benefit in reducing the triglyceride (TG) values.

## 1. Introduction

The number of women entering menopause has increased worldwide due to the increment of life expectancy. One of the important issues affecting postmenopausal women is the risk and benefits of estrogens replacement therapy. Many of the women who are prescribed hormone replacement therapy stop taking it or never commence taking it due to fear of associations with malignancy, unacceptable bleeding, or its side effects. Instead, they search for alternative ways of self-managing the postmenopausal symptoms or its long term consequences, such as increased risk of coronary artery disease (CAD) and osteoporosis.

*Labisia pumila *(LP) or more commonly known as Kacip Fatimah has been used widely in South East Asian communities for a variety of illnesses and in food supplements. This plant has been widely used by Malaysian women for generations to ease childbirth and also for its postpartum rejuvenating properties [1]. Traditionally, LP extract is prepared by boiling the roots, leaves, or the whole plant in water, and the extract is consumed orally [1]. Its exclusive use by women has led to the belief that it is a phytoestrogen, a compound with similar chemical structures to estrogen [2], and is therefore able to relieve menopausal symptoms [3].

*L. pumila* is from the genus Myrsinaceae. Three different varieties of *L. pumila* were identified in Malaysia: var*. alata, *var.* pumila*, and var.* lanceolata* [4]. The term Kacip Fatimah is used to describe the plant in general. Preliminary studies have shown that the var.* alata* and var.* pumila* are more commonly used medicinal plants than var.* lanceolata.*

Phytochemical studies of the roots and leaves of *L. pumila* var.* alata* have shown the presence of three C_15_ monoene resorcinols that are (Z)-5-(pentadec-4-enyl) benzene-1-3,3-diol, (Z)-5-5-(pentadec-8-enyl) benzene-1-3,3-diol, and (Z)-5-5-(pentadec-10-enyl) benzene-1-3,3-diol [5]. In addition, it also contains two novel benzoquinoid compounds 1, 2 as major components [6].

Research has demonstrated the estrogenic activity of LP. It is possible that it acts as selective estrogen receptor modulators (SERMs) which is active in certain tissues, [7]. A study showed that water extracts of LP were able to displace estradiol binding to antibodies raised against estradiol, making it similar to other estrogens such as estrone and estradiol [7]. The extract has also been found to produce a dose-response effect on the reproductive hormones of female rats, notably on the estradiol and free testosterone levels [7].

Recently, study has showed that *Labisia pumila* (LP) is a potential alternative agent for hormone replacement therapy in postmenopausal women [8]. In that study, a group of researchers [8] had conducted a study comparing the effect of LP aqueous extract to estrogen on reproductive hormones using ovariectomised rat model. *Labisia pumila* supplementation had been shown to resemble the effect of estrogen replacement therapy on reproductive hormones [8]. In the study, it showed that 60-day treatment with LP significantly reduced luteinizing hormone (LH) and follicle stimulating hormone (FSH). It also elevates the estradiol and testosterone levels. These results resembled the effect of estrogen in the ovariectomised rats.

Currently, *L. pumila *is manufactured in the form of tonics or capsules by local companies, and various claims have been made that *L. pumila* can improve the well-being of women. It is imperative that all these declarations regarding the benefits and advantages of *L. pumila *or any herbal formulations containing *L. pumila *are proven scientifically, and that any possible toxicity arising from its consumption is evaluated.

In view of the initial evidence, it is postulated that this plant has a beneficial effect on postmenopausal women in terms of the positive impact on the lipids and hormonal profiles. The present study was designed to investigate the effect of LP on menopausal symptoms, cardiovascular risk factors, and hormonal profiles in Malay postmenopausal women.

## 2. Materials and Methods

### 2.1. Study Design and Setting

This randomised, parallel-group, placebo-controlled study to compare the effects of *Labisia pumila *var.* alata *extract at 280 mg/day was carried out amongst postmenopausal Malay women at the Clinical Trial Unit, Hospital Universiti Sains Malaysia (HUSM) from June 2004 until May 2005.

### 2.2. Study Participants

Malay women aged between 48 to 55 years with the body mass index ranging from 18 to 35 kg/m^2  ^were eligible for the study if they had been postmenopausal for at least six months. The women were not assessed on whether they had any menopause symptoms. The exclusion criteria included a history of taking hormone replacement therapy or any herbal products for at least 6 months, a history of oophorectomy, a history of alcohol or drug abuse, a history of breast or cervical cancer, any active medical illnesses making the implementation of the study protocol or interpretation of the results difficult, or the presence of endometrium thickness of more than 0.5 cm detected with a pelvic ultrasonography.

The study participants were identified from the Obstetrics & Gynaecology Clinic or from the outpatients' clinic of HUSM. They were invited to come to the Clinical Trial Unit for the study explanation and screening procedure. Informed consent was obtained once they agreed to participate in the study. A detailed history including past medical history and gynaecology history was assessed at baseline. The physical examination including blood pressure, weight, height, and waist hip ratio measurement, and cardiovascular, breast, abdomen, and pelvic examinations including Pap smear and pelvic ultrasound were performed at the initial and the last visit.

### 2.3. Randomization and Interventions

Study participants were randomly allocated to either LP or placebo by means of computer-generated randomization numbers issued by the Institute for Medical Research (IMR). Subject eligibility was established before treatment randomization which was done after the baseline visit and after the patient agreed to participate and signed the informed consent. The research nurse determined the treatment allocation by drawing a sealed nonopaque envelope containing instructions on the treatment allocations.

### 2.4. Plant Material

The raw material of *Labisia pumila* var.* alata* was identified and authenticated by ethno botanist of the Forest Research Institute of Malaysia (FRIM). The preparation of water extract of *Labisia pumila* var.* alata *is done by subjecting the dried plant material to water to form a water-soluble extract and then desiccating the extract. The process for preparation of *Labisia pumila *extract is by extracting dried *Labisia pumila *plant material with water at a ratio of 1 : 6 of dried *Labisia pumila *plant material:water to form a water-soluble extract and drying the extract wherein the extracting is carried out at 80°C for 3 hours and with continuous stirring. The starting material is fully dehydrated by drying it at 40°C for three days. The process wherein the extracting is repeated and the ratio of *Labisia pumila *plant material:water is 1 : 6. Then the *Labisia pumila *extract is dried by spray drying and wherein the spray drying comprises concentrating and drying. The spray drying is performed using a spray tower having a tower inlet and outlet, wherein tower inlet temperature is 185°C, and wherein tower outlet temperature is 107°C, respectively. Then a process for isolating a marker compound by semipreparative reverse-phase high-performance liquid chromatography was done from *Labisia pumila *extract. The marker compound is 3,4,5-trihydroxybenzoic acid.

The extract that was used in this study was similar with most of the animal studies [7–10] conducted since it was a big research project planned by the Malaysia government under the grant provided by the Ministry of Science, Technology and Innovation (MOSTI). *Labisia pumila *var.* alata* extract was prepared and packed in a sachet form by a Good Manufacturing Practice (GMP) certified herbal company that has been approved by the Drug Control Authority, Ministry of Health, Malaysia.

### 2.5. Dosage and Study Protocol

The dosage used was based on animal studies carried out by the Institute for Medical Research Malaysia (IMR). Based on the study, a significant response in estradiol and testosterone levels were seen when the rats received 16 mg/kg body weight of *L. pumila *var.* alata *extract. Using the conversion dose as suggested by Freireich et al. [11] where the dose conversion used was 1/7 of rat dose equals to a 60 kg man dose, the minimum dose of *L. pumila *var.* alata* extract to be tested was 140 mg. In this study, we used the double dose of the minimum dose needed because we did not know the dose that will give significant response in human. Due to budget constraint to do study for all three doses that is 140 mg, 280, or 560 mg, we decided to use 280 mg of *L. Pumila* extract per day for this study.

The study subjects were required to take two sachets of the extract of *L. Pumila *daily at night for six months. The compliance was measured using the numbers of sachets taken. The sachets were supplied to the patients every month, and subjects were asked to return all unused medication. The number of sachets issued minus the number of sachets returned was used to calculate the compliance.

### 2.6. Outcome Measures and Followups

The women were given questionnaires to assess their menopausal symptoms. This questionnaire was validated and based on previous study [12]. It consisted of a list of symptoms which comprised of classical vasomotor symptoms, physical symptoms, and psychological symptoms. Each of the symptoms was assessed using the Likert scale from 0 to 5. They were asked to fill in this questionnaire at baseline and at the end of six months. Blood samples to measure fasting glucose levels, total cholesterol, low density lipoprotein (LDL), high density lipoprotein (HDL), triglycerides, luteinizing hormone (LH), follicle stimulating hormone (FSH), and estradiol levels as study outcomes were taken at baselines and at two-month intervals. The subjects were followed up every two months for the total duration of six months. At each visit, the subjects were examined by medical specialists who were part of the clinical trial team, and they were asked for feedback regarding any side effects.

### 2.7. Approval by the Research and Ethics Committee

Ethical approval was obtained from the Research and Ethics Committee, Universiti Sains Malaysia prior to the study implementation. The protocol was approved by the Research and Ethical Committee, School of Medical Sciences, University Sains Malaysia (USM/PPSP/Ethics Com./2004 (125.4(6)).

### 2.8. Statistical Analyses

Analyses were done by using SPSS for windows version 12.0, and all numerical variables were expressed as mean and standard deviation (SD) and categorical data as frequencies and percentages. Randomized groups were compared for any possible differences at baseline using independent *t*- and chi-square tests. To show the effectiveness of the treatment group, analysis of covariance (ANCOVA) was used to compare the difference of the outcome variables at 6-month postintervention after controlling the age, BMI, duration of menopause, and the baseline values as the covariates. Summary changes were reported as means, standardizing changes for possible covariate imbalances between groups. All reported *P* values are 2-tailed with a value of less than 0.05 which is considered significant.

## 3. Results

A total of 63 patients completed the 6-month study. The baseline characteristic of the study participants is shown in Table 1. There were no statistical significant differences between the treatment group and placebo at baseline in terms of age, duration of menopause, parity, clinical, biochemical, and hormonal parameters.

### 3.1.  Table 2

It displays the mean of the menopausal symptoms scores of both groups comparing the baseline and the last visit. Both groups showed a reduced trend within the groups. However, there was no significant difference observed between the placebo and the verum (treatment) group.

### 3.2.  Table 3

It shows the cardiovascular risk factors and hormonal changes after the 6-month followup trial with placebo and LP at 280 mg. After adjustments were made regarding age, BMI, duration of menopause, and its baseline values, there was a significant main effect of treatment on the TG levels [*F*(1,63) = 4.309, *P* = 0.042, ANCOVA] where the adjusted mean of TG in LP subjects was significantly lower than placebo (1.4 versus 1.9 mmol/L) (adj. mean difference 0.5, 95% CI: 0.02, 0.89) (Table 3). There were also comparatively lower means of the fasting plasma glucose and total cholesterol in the LP treatment group; however, the differences were not statistically significant.

## 4. Discussion

Previously, hormone replacement therapy has subsisted as the mainstay of treatment for problems arising from loss of ovarian functions. However, recent studies have changed this practice due to adverse effects as regards to hormone replacement therapy (HRT) [13]. Now, there are many controversial issues regarding prescribing HRT for postmenopausal women. These is a cause for the tremendous growth in the use of alternative therapies to relieve the postmenopausal symptoms. Many women assume that alternative medicine is safe and natural. However, compared to HRT, there are still not sufficient clinical trials to assess the effect of phytoestrogen on cardiovascular risk factors and osteoporosis evidenced by changes in bone mineral density.

There is still very little information about *Labisia pumila* var.* alata *chemical properties and the mechanism of action despite its wide range of use among females in Malaysia. The plant root and leaves were found to contain two novel benzoquinoid compounds 1, 2 as major components [6]. Another group of researcher had conducted a study which looked at the adverse effects of the aqueous extract of this plant on the oestrous cycle, reproductive performance, postnatal growth, and the offsprings of rats [9]. In that study, the water-based extracts did not pose any significant reproductive toxicity or complication during pregnancy and delivery in rats. Currently, to our knowledge, the toxicology studies were done only in rats.

Theoretically, phytoestrogens exert their effects primarily through binding to estrogen receptors (ERs) [14]. Reports have shown that the plant displays a nonsignificant response to *in vitro* estrogen activity [5]. The water extract of LP was shown to be able to displace estradiol binding to antibodies raised against estradiol making it similar to other estrogens such as estrone and estradiol [7]. The extract also produced a dose response effect on the reproductive hormones of female rats, notably on estradiol and free testosterone levels [7]. However, in this study, there was no effect of LP on the menopausal symptoms and hormonal profiles in the post menopausal women. The possible explanations are that the sample size in this study is very small, and also it needed to be given in a longer duration before the effects can be seen. In animal study, it showed that estrogen replacement therapy increased estradiol level as early as 30 days of treatment compared to LP which requires 60 days of treatment [8].

There were few limitations in this study. Although the treatment group showed reduced triglyceride levels compared to the placebo group, however the level in both groups was still within the normal range. Therefore, it is of no clinical relevance at this moment. We suggest that further study is required in order to assess this effect. In an animal study, it was revealed that the ovariectomised rats treated with LP extract had decreased in body weight compared to the control group and have postulated that this mechanism occurred due to the presence of phytoestrogen in it [10]. The study postulates that there is a possible role for *Labisia pumila* var.* alata* in modulating postmenopause adiposity in a manner similar to that reported for estrogen through the initiation of the lipolysis process in adipose tissue and thus may have a possible effect on weight management [10].

In the other study [15], which looked at the effect of water extract from *Labisia pumila* on the aorta of ovariectomized rats, it was found that the elastic lamellae architecture of the ovariectomized rat aortae in the treatment group by the plant was maintained in a manner comparable to the normal rat. This result implied that there is a possible role for LP in modulating postmenopausal cardiovascular risks.

This study involved a small sample size which may have had an impact on why some of the results might not be significant. Conducting a bigger study is highly recommended in order to look at the cardiovascular effect of LP and probably its weight reduction effect.

In conclusion, the result showed that *Labisia pumila* has beneficial effect to reduce the triglyceride (TG) values. Thus, it may be a useful phytosupplement for maintaining cardiovascular health in menopausal women. However, there was no effect on the hormonal profiles.

## Figures and Tables

**Table 1:** Baseline characteristics among participants of randomized controlled trial between *Labisia pumila* and placebo groups.

Characteristics	All* (*n* = 63)	Trial groups*		*P* value^‡^
		*Labisia * (*n* = 29)	Placebo (*n* = 34)	
Age (years)	52.7 (1.9)	52.9 (1.8)	52.6 (1.9)	0.445
Age at menarche (years)	13.9 (1.7)	13.9 (1.7)	13.9 (1.7)	0.790
Duration of menopause (years)	3.2 (3.1)	3.0 (2.9)	3.6 (3.2)	0.495
Number of parity (%)^†^				
<5	23 (36.5)	8 (27.6)	15 (44.1)	0.174
≥5	40 (63.5)	21 (72.4)	19 (55.9)	
Household income per month (%)^†^				
≤RM 1,000	39 (61.9)	19 (65.5)	20 (58.8)	0.586
>RM 1,000	24 (38.1)	10 (34.5)	14 (41.2)	
Body mass index (BMI) (kg/m^2^)	26.5 (3.7)	26.4 (4.5)	26.5 (3.1)	0.814
Waist hip ratio	0.85 (0.08)	0.85 (0.08)	0.86 (0.09)	0.812
Diastolic BP (mm Hg)	82.9 (10.1)	81.8 (10.5)	83.8 (9.9)	0.439
Systolic BP (mm Hg)	125.3 (18.5)	122.8 (18.5)	127.3 (18.4)	0.339
Total cholesterol (mmol/L)	5.2 (1.1)	5.1 (1.1)	5.3 (1.1)	0.601
Triglycerides (mmol/L)	1.7 (1.4)	1.4 (1.1)	1.9 (1.6)	0.095
Fasting glucose (mmol/L)	6.1 (1.9)	5.8 (0.7)	6.2 (2.6)	0.433
Female hormones (IU)				
FSH	49.0 (22.2)	45.8 (23.7)	51.8 (20.8)	0.297
LH	23.6 (13.6)	23.8 (14.8)	23.3 (12.6)	0.877
Estrogen	38.7 (19.0)	35.7 (12.2)	41.2 (22.9)	0.271

*Values are expressed as mean (standard deviation, SD) unless otherwise specified.

^‡^Determined by independent *t*-test; ^†^chi-square tests.

**Table 2:** The menopausal symptoms changes after 6-month followup trial with placebo and *Labisia pumila. *

Menopausal symptoms	*Placebo (*n* = 34)	*Labisia (*n* = 29)	*P* value^‡^
Hot flushes	V1 0.96 (1.34)	V1 1.10 (1.32)	0.75
	V2 0.56 (0.93)	V2 0.90 (1.49)	
Night sweats	V1 1.04 (1.43)	V1 1.24 (1.30)	0.49
	V2 0.59 (1.04)	V2 0.79 (1.17)	
Insomnia	V1 1.56 (1.60)	V1 1.31 (1.58)	0.78
	V2 0.89 (1.15)	V2 0.90 (1.34)	
Dyspareunia	V1 1.89 (1.91)	V1 1.72 (1.62)	0.93
	V2 1.37 (1.49)	V2 1.59 (1.66)	
Painful joints	V1 2.56 (1.72)	V1 2.66 (1.57)	0.63
	V2 1.33 (1.44)	V2 2.03 (1.38)	
Mastalgia	V1 0.56 (1.25)	V1 0.17 (0.38)	0.52
	V2 0.19 (0.48)	V2 0.21 (0.62)	
Palpitation	V1 1.48 (1.55)	V1 0.86 (1.56)	0.19
	V2 0.52 (0.80)	V2 0.55 (0.95)	
Irritability	V1 2.15 (1.59)	V1 2.34 (1.54)	0.34
	V2 1.07 (1.17)	V2 1.54 (1.21)	
Difficulty in concentration	V1 1.26 (1.58)	V1 2.07 (1.41)	0.16
	V2 1.15 (1.17)	V2 1.79 (1.37)	
Memory problem	V1 1.89 (1.50)	V1 2.07 (1.41)	0.52
	V2 1.15 (1.17)	V2 1.79 (1.37)	
Lethargic	V1 2.04 (1.69)	V1 1.59 (1.59)	0.75
	V2 0.96 (1.29)	V2 0.97 (1.05)	

*Values are expressed as mean (standard deviation, SD) unless otherwise specified.

^‡^Determined by independent *t*-test; ^†^chi-square tests.

**Table 3:** Cardiovascular risk factors and hormonal changes after 6-month followup trial with placebo and *Labisia pumila* at 280 mg.

Study variables	Trial groups^†^			*P* value^‡^
	Placebo (*n* = 34)	*Labisia * (*n* = 29)	Adjusted mean difference (95% CI)	
Cardiovascular disease risk factors				
Total cholesterol (mmol/L)	5.5 (5.2, 5.9)	5.0 (4.6, 5.4)	0.5 (−0.06, 1.04)	0.082
Triglycerides (mmol/L)	1.9 (1.6, 2.2)	1.4 (1.1, 1.7)	0.5 (0.02, 0.89)	0.042
Fasting glucose (mmol/L)	6.3 (5.8, 6.8)	5.6 (5.1, 6.2)	0.7 (−0.06, 1.38)	0.073
Diastolic BP (mm Hg)	81.2 (78.3, 84.1)	82.1 (78.9, 85.2)	0.9 (−3.4, 5.2)	0.686
Systolic BP (mm Hg)	125.8 (120.9, 130.7)	127.5 (122.1, 132.8)	1.7 (−5.6, 9.0)	0.643
Female hormones (IU)				
FSH	36.3 (29.7, 42.8)	38.4 (31.3, 45.5)	2.11 (−7.62, 11.84)	0.665
LH	26.2 (21.8, 30.5)	24.4 (19.9, 29.0)	1.75 (−4.59, 8.08)	0.583
Estrogen	37.5 (31.4, 43.5)	33.5 (26.7, 40.3)	3.94 (−5.23, 13.10)	0.393

Abbreviations: LH: luteinizing hormone; FSH: follicle stimulating hormone; BP: blood pressure.

^†^Values at 6-month followup are expressed as adjusted means (95% confidence interval, CI) and are adjusted for baseline values, age, BMI, and duration of menopause.

^‡^Determined by analysis of covariance (ANCOVA); *P* value < 0.05 is considered significant.
